# Finding a Diagnosis in Obscure Gastrointestinal Bleeding: A Case of Small Bowel Neuroendocrine Tumor

**DOI:** 10.7759/cureus.95034

**Published:** 2025-10-21

**Authors:** Lin Deng, Rahul Vasavada, Christofer Bello

**Affiliations:** 1 Internal Medicine, University of California Los Angeles, Los Angeles, USA; 2 Department of Pathology and Laboratory Medicine, David Geffen School of Medicine at University of California Los Angeles, Los Angeles, USA

**Keywords:** double balloon enteroscopy, obscure gi bleeding, small bowel gi bleeding, small bowel neuroendocrine tumor, video capsule endoscopy

## Abstract

Small bowel or mid-gut gastrointestinal (GI) bleeding has been historically difficult to diagnose and is believed to be the main culprit of what was once termed obscure GI bleeding. Small bowel bleeding is difficult to evaluate due to its inaccessibility by direct visualization. Due to recent innovations in diagnostic techniques such as video capsule endoscopy (VCE), deep enteroscopy, and CT enterography, the capability to visualize and diagnose pathologies within the small bowel has improved. One such pathology is neuroendocrine tumors (NETs), which in recent years have overtaken adenocarcinoma as the most common malignancy of the small intestine. Early-stage small bowel neuroendocrine tumors (SBNETs) in the jejunal-ileum are difficult to detect due to their indolent nature and are often found only after they metastasize. We describe a case of small bowel GI bleeding in a functional 90-year-old patient where methodological use of advanced diagnostic modalities with video capsule endoscopy and double balloon enteroscopy helped identify an early-stage SBNET of the jejunal-ileum, leading to timely surgical intervention.

## Introduction

Small bowel bleeding, or mid-GI bleeding, refers to bleeding that originates between the ligament of Treitz and the ileocecal valve. It is the least common form of GI bleed accounting for 5-10% of total GI bleed cases [1], but is thought to be the most common cause of obscure GI bleeding. Malignancy is an uncommon but important cause of mid-GI bleed, as early detection can lead to potentially life-saving treatment. According to data from the Surveillance, Epidemiology, and End Results (SEER) program, neuroendocrine tumor (NET) is the most common malignancy of the small bowel accounting for 37.4% of cases [2]. Between 1973 and 2012, small bowel neuroendocrine tumors (SBNETs) have had a 6.4-fold increase in incidence (from 1.09 to 6.98 per 100,000 persons [3,4]). Despite this increase in incidence, diagnosing SBNETs remains challenging as majority of patients with early disease are either asymptomatic or have subtle, nonspecific symptoms such as abdominal pain, obstruction, and/or GI bleeding. In addition, direct visualization of the small bowel is difficult by traditional esophagogastroduodenoscopy (EGD) and colonoscopy. Recent advancements in diagnostic imaging and endoscopy have allowed clinicians to better identify etiologies of small bowel bleeding including SBNETs.

## Case presentation

A 90-year-old male with past medical history of aortic stenosis, bioprosthetic aortic valve replacement, hyperlipidemia, and remote history of peptic ulcer disease presented with one week of black stools associated with increasingly worsening fatigue and dyspnea on exertion. The patient was previously able to walk multiple blocks without issue and was living independently at home. A comprehensive review of systems was otherwise negative except for nasal congestion and non-productive cough. He was not aware of any family history of gastrointestinal disease or malignancy. He drank half a glass of wine and a beer every night but never smoked. His home medications included baby aspirin and atorvastatin daily. He denied taking any other blood thinners or non-steroidal anti-inflammatory drugs (NSAIDs). On admission, the patient’s vital signs were within normal limits. His initial labs showed normocytic anemia, with a hemoglobin level that was acutely low at 6.8 g/dL (Table 1). 

**Table 1:** Admission lab values WBC: White blood cells; MCV: mean corpuscular volume; BUN: blood urea nitrogen; AST: aspartate aminotransferase; ALT: alanine aminotransferase; ALP: alkaline phosphatase

Lab Test	Value	Reference Range
Complete Blood Count		
WBC	11.08 k/UL	4.00-11.00 k/UL
Hemoglobin	6.8 g/dL	13.0-17.0 g/dL
Hematocrit	20.6 %	37.5-49.9%
MCV	98.1 FL	80.0-100.0 FL
Platelet count	222 k/UL	150-450 k/UL
Basic Metabolic Panel		
Sodium	133 mmol/L	132-146 mmol/L
Potassium	3.9 mmol/L	3.5-5.0 mmol/L
Chloride	106 mmol/L	98-107 mmol/L
Carbon Dioxide	24 mmol/L	22-31 mmol/L
Anion Gap	7 meq/L	10-20 meq/L
BUN	12.1 mg/dL	8.4-25.7 mg/dL
Creatinine	0.66 mg/dL	0.72 – 1.25 mg/dL
Liver function Tests		
Albumin	2.8 g/dL	3.2-4.6 g/dL
AST	24 U/L	5-34 U/L
ALT	12 U/L	0-55 U/L
ALP	36 U/L	40-150 U/L
Direct Bilirubin	0.3 mg/dL	0.0-0.5 mg/Dl
Indirect Bilirubin	1.6 mg/dL	<1.0 mg/dL
Total bilirubin	1.9 mg/dL	0.2-1.2 mg/dL
Total Protein	4.5 g/dL	6.4-8.3 g/dL

**Figure 1:**
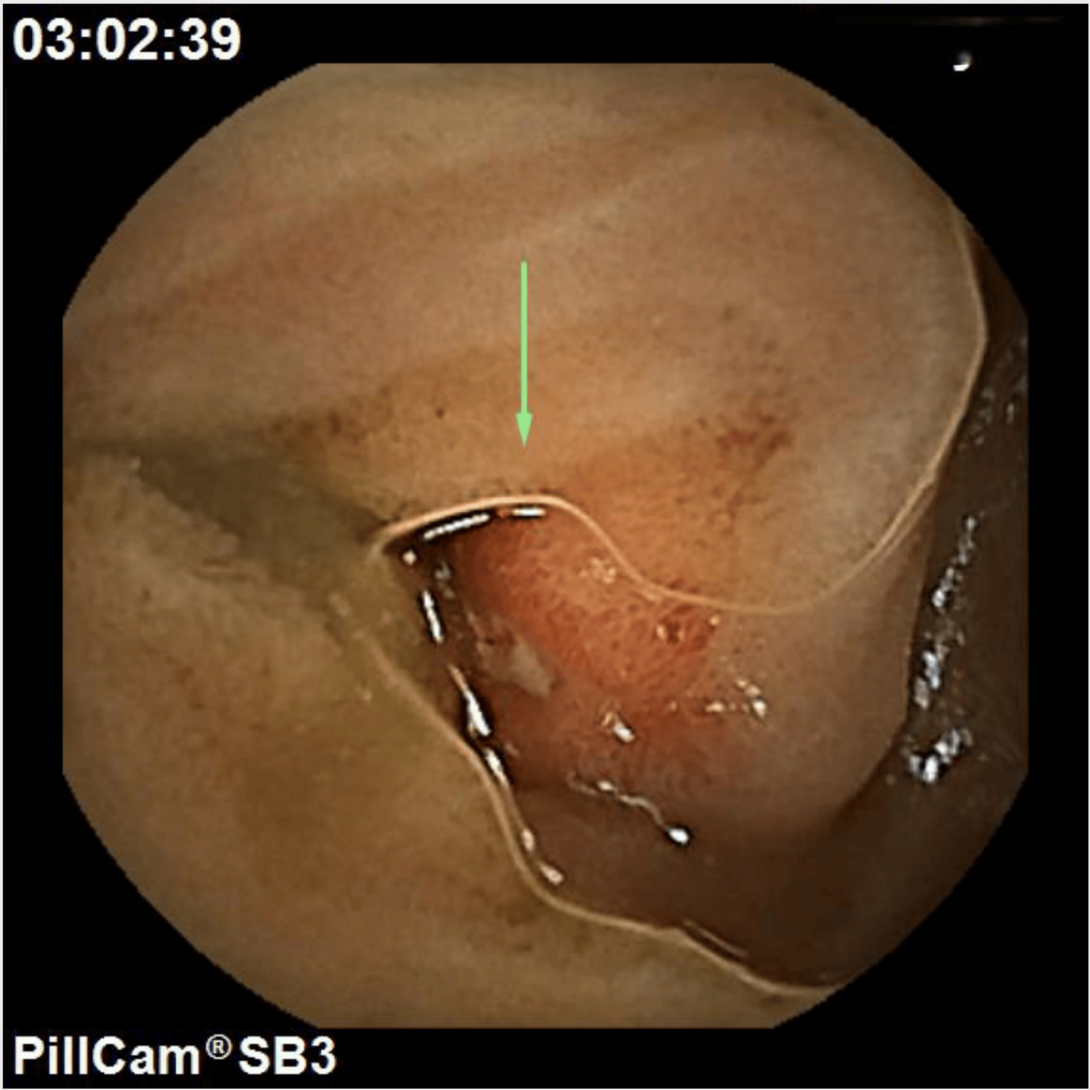
Video capsule endoscopy. Erythematous mucosa with central ulceration with pigmented spot at 39% small bowel transit that is likely the source of bleeding (arrow).

He received one unit of packed red blood cell (PRBC) transfusion, but hemoglobin continued to downtrend to 6.0 g/dL (reference range 13.0-17.0 g/dL). He was started on intravenous (IV) proton pump inhibitor and IV iron supplementation. A second unit of PRBC transfusion was given. EGD performed by gastroenterology showed a non-bleeding gastric ulcer with a clean base, and a duodenal adenoma without ulceration. It was felt that the EGD findings did not explain the patient’s degree of anemia, so a colonoscopy was pursued. This revealed pan-diverticulosis and maroon blood in the ileum 40-60cm from the ileocecal valve with more blood seen proximally, which was concerning for small bowel bleeding. A wireless video capsule endoscopy (VCE) was done next, which showed evidence of bleeding at 39% of small bowel transit with ulceration and blood distally (Figure 1).

**Figure 2:**
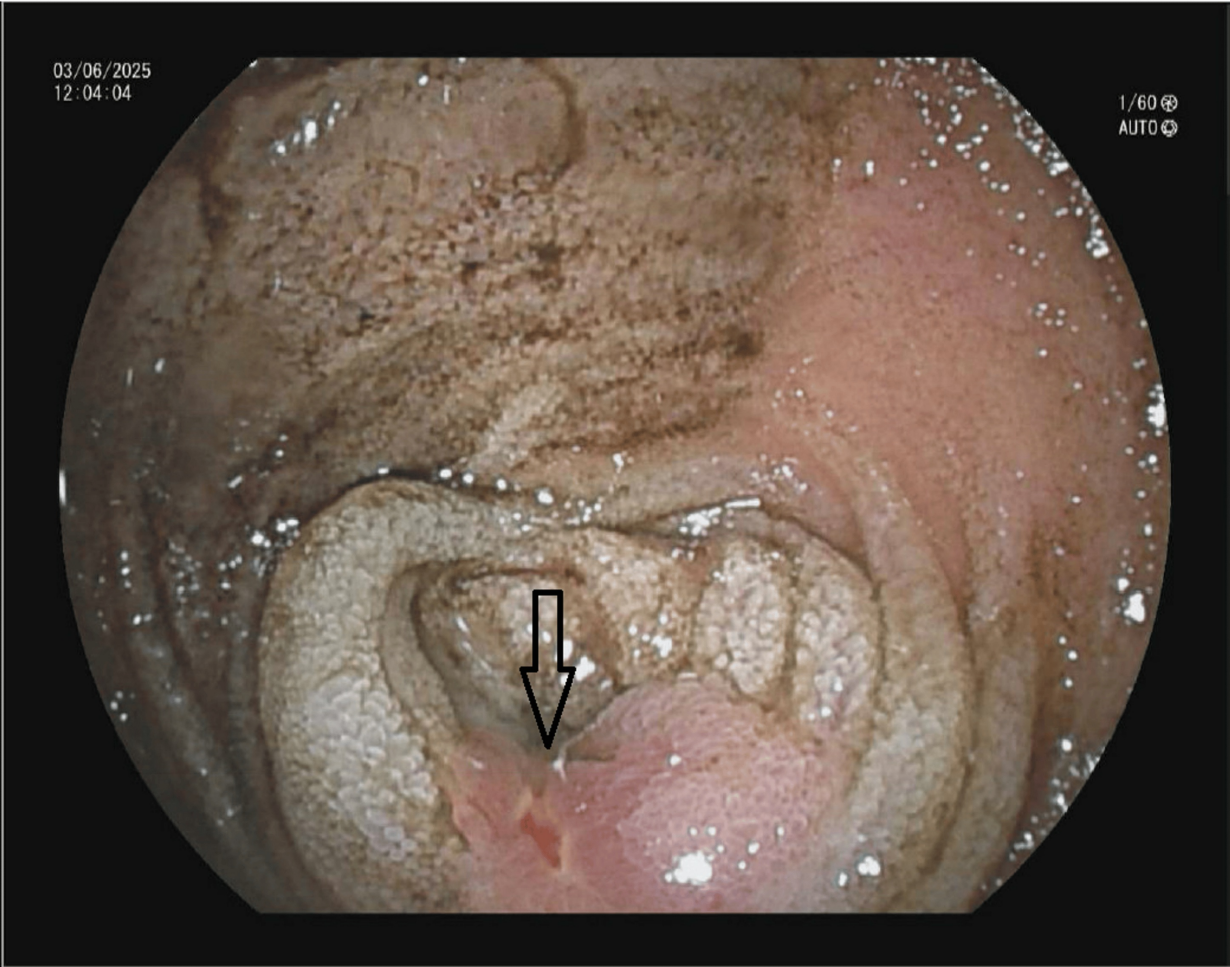
Small intestinal double balloon enteroscopy. Ulcer with luminal narrowing suggestive of a submucosal eroding lesion (arrow).

Given these findings, an antegrade double balloon enteroscopy (DBE) was performed and revealed a 15mm ulcerated lesion with some narrowing in the proximal ileum (Figure 2).

The area was marked, and biopsy of the ulcerated lesion showed a well-differentiated, grade 1 neuroendocrine tumor in the small intestinal mucosa. The Ki-67 proliferation index was <1%, and the mitosis count was <1 per 2mm^2^. Surgical oncology was consulted. Staging computed tomography (CT) scans of the chest, abdomen, and pelvis revealed a partially calcified mid-mesenteric nodule that could represent metastasis, but no liver or intrathoracic metastases were observed. By this point, the patient’s GI bleeding had ceased, and his hemoglobin stabilized at around 8.0 g/dL (reference range 13.0-17.0 g/dL) after a total of three units of PRBC transfusions. Due to clinical stability, insurance issues, and patient preference, the patient was discharged with close outpatient follow-up with his in-network providers for further surgical planning.

During the patient's follow-up visit with surgery and oncology, additional laboratory workup showed iron deficiency anemia with normal folate and vitamin B12 levels (Table 2). Biomarkers for SBNETs such as chromogranin-A and serotonin were within normal limits. 5-hydroxyindoleaetic acid (5-HIAA) levels were not measured. 

**Table 2:** Additional hematologic studies and tumor markers N/A: Not applicable

Lab Test	Value	Reference Range
Iron	18 mcg/dL	41-179 mcg/dL
Iron Binding Capacity	302 mcg/dL	262-502 mcg/dL
% Saturation	6 %	N/A
Ferritin	83 ng/mL	8-350 ng/mL
Folate	14.0 ng/mL	8.1-30.4 ng/mL
Vitamin B12	411 pg/mL	254-1060 pg/mL
Chromogranin-A	161 ng/mL	0-187 ng/mL
Serotonin	127 ng/mL	50-220 ng/mL

**Figure 3:**
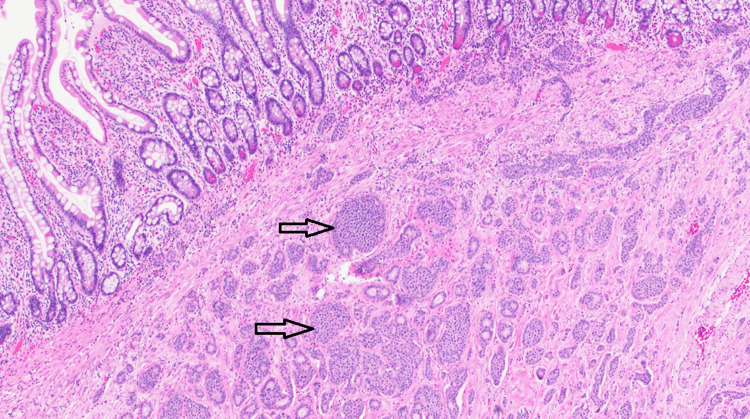
Histologic section of the patient’s small bowel mass shows a well differentiated neuroendocrine tumor with cells arranged in a nested to insular pattern (arrows). A low mitotic count (< 2 mitoses per 2 mm2) and a low Ki67 proliferation index (< 3%) classifies this tumor as grade 1.

The patient then underwent somatostatin receptor imaging with positron emission tomography (PET)/CT of the neck, chest, abdomen, and pelvis. This showed an enhancing mass in the ileum consistent with biopsy confirmed SBNET, with an avid mesenteric mass in the lower-mid abdomen compatible with nodal metastasis. Given the patient’s good functional status, localized spread of the tumor, and symptomatic anemia requiring hospitalization, the decision was made to proceed with elective laparoscopic resection of the small bowel along with the mesenteric lesion. Subsequent surgical pathology of the resected small bowel mass revealed a well differentiated grade 1 NET (Figure 3). 

**Figure 4:**
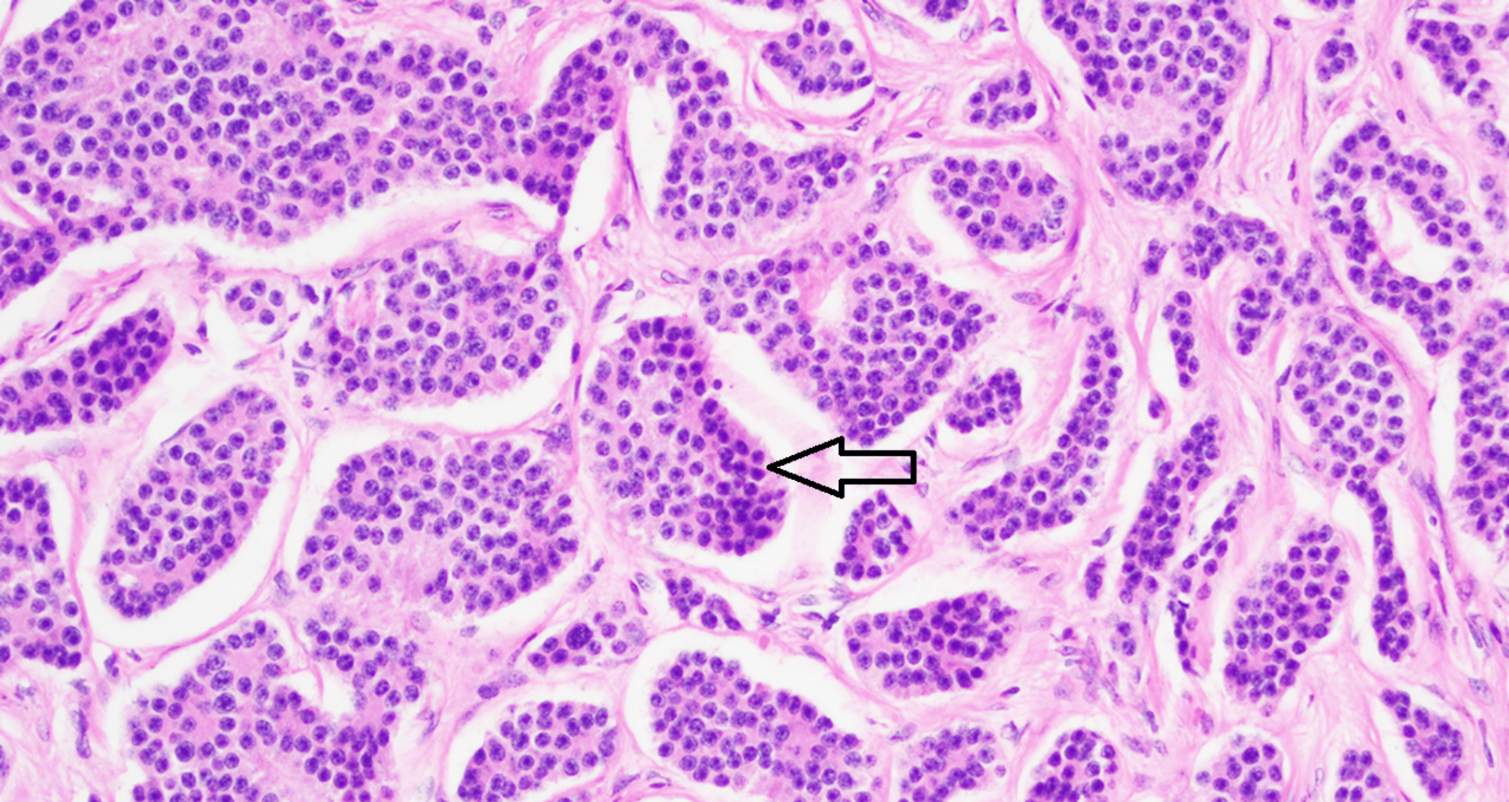
A high power view of the mesenteric mass shows similar morphology to the small bowel mass. The monotonous cells show round to ovoid nuclei with “salt and pepper” chromatin and moderate eosinophilic granular cytoplasm (arrow).

The resected mesenteric mass showed a similar morphology consistent with a locoregional metastasis (Figure 4). The patient’s postoperative course was complicated by ileus requiring bowel rest, but he was ultimately discharged in good condition. His hemoglobin remained stable without signs of further bleeding during his postoperative follow-up, and no adjuvant therapy was planned.

## Discussion

The term obscure GI bleed refers to bleeding where no source is identified on EGD and colonoscopy, but evidence of GI bleeding persists via fecal occult blood test positivity, continued iron deficiency anemia, and/or visible blood loss. With recent advances in small bowel diagnostics, the American College of Gastroenterology (ACG) proposed to reclassify obscure bleeding as small bowel bleeding since the majority (~75%) of culprit lesions are being identified in the small bowel [5]. The etiology of small bowel bleeding differs slightly based on age. In the elderly population (>60 years old), angiodysplasia, ulcers, and malignancies are more common, whereas inflammatory bowel disease (IBD), Dieulafoy lesions, and Meckel’s diverticulum are usually seen in younger patients [1,5].

The patient in our case presented with normocytic anemia and symptoms suggestive of melena. Although melena usually portends bleeding from an upper GI source, small bowel and slow right-sided colonic bleeding can also present similarly [6]. In cases of recurrent clinical bleeding, a second-look EGD or colonoscopy is often considered to exclude an initially missed lesion, and has been shown to have a positive diagnostic yield of up to 29% and 23%, respectively [5,7]. When both EGD and colonoscopy fail to reveal an actively bleeding lesion despite worsening anemia, the diagnosis of obscure or small bowel bleeding becomes high on the differential. As in our patient’s case, since maroon blood was visualized proximal to the ileocecal valve, a small bowel evaluation was pursued with video capsule endoscopy.

Since its advent in 2001, VCE has become the test of choice for the evaluation of suspected small bowel bleeding. It has a diagnostic yield ranging from 38-83%, and high positive (94-97%) and negative predictive values (82.6-100%) [5,8]. Metanalysis comparing VCE to push enteroscopy and small bowel barium radiography have shown an incremental yield (30% and 36%, respectively) for clinically significant findings in small bowel bleeding [9]. There is also evidence that early use of VCE (within 48-72 hours of small bowel bleeding) has the greatest yield for detecting a bleeding source, with some studies reporting 64% bleed localization in the early VCE arm versus 31% in the standard arm [10]. One caveat, however, is that VCE often fails to detect the major papilla in the duodenum and should not be relied upon as the only test to detect pathology in this region. The main complication of VCE is capsule retention, and the study should be avoided in favor of CT enterography if there is concern for bowel obstruction [5].

Double balloon enteroscopy is often used diagnostically and therapeutically in small bowel bleeding, as it can travel deeper into the small bowel compared to traditional EGD and colonoscopy. While multiple studies comparing VCE with DBE have shown similar diagnostic yield, VCE is often done prior to DBE to help localize the lesion and guide subsequent DBE approach (antegrade vs retrograde) [11]. Using VCE as a screening tool before DBE is not only safer and more tolerable for patients, but this complementary approach has been shown to increase the diagnostic (73-93%) and therapeutic yield (57-73%) of DBE [12]. In our case, early use of VCE after initial EGD and colonoscopy was critical in identifying the culprit lesion in the proximal ileum - allowing for timely biopsy and marking by DBE followed by curative resection. The main limitations of DBE are its longer procedure times and higher complication rates, especially with perforation compared to standard endoscopy [13].

NETs are rare, slow-growing tumors accounting for 0.5% of all cancers and two percent of all GI malignancies. SBNETs have seen an increase in incidence and prevalence over the last few decades in part due to increased awareness and adoption of advanced endoscopic and imaging techniques [14]. Neuroendocrine tumor cells contain chromogranin-A, synaptophysin, and neuron-specific enolase and can secrete hormones. They are graded based on the mitotic count and the proportion of Ki-67 positive tumor cells (Ki-67 index) correlating with cellular proliferation [15].

Clinically, jejunal-ileal SBNETs are often small and asymptomatic until they cause nonspecific symptoms such as abdominal pain, bowel obstruction, or GI bleeding [4,15]. The classic carcinoid syndrome with flushing, diarrhea, and/or wheezing only develops in 20-30% of the patients when the tumor metastasizes to the liver [14]. Risk factors for SBNET development are still poorly understood. Some studies suggest a positive correlation between SBNET development and smoking, as well as a history of prostate or colorectal cancer in the patients’ first-degree relatives [15]. Our patient did not have any notable risk factors for SBNET. His symptoms were mostly fatigue and GI bleeding. He did not exhibit any signs of carcinoid syndrome.

Biomarkers such as chromogranin-A and 5-HIAA are often used to guide diagnosis and monitor treatment response. Chromogranin-A is a polypeptide highly sensitive to functioning and non-functioning SBNETs, but can be falsely elevated in renal impairment or patients taking proton pump inhibitors. Urine or serum 5-HIAA measures the breakdown product of serotonin that is secreted by carcinoid tumors and is often used to monitor patients with suspected carcinoid syndrome and to assess treatment response [4,15].

Staging of SBNETs usually involves initial triple phase CT or magnetic resonance imaging (MRI) of the chest, abdomen, and pelvis with contrast, followed by somatostatin receptor PET/CT or PET/MRI. Distal SBNETs in the jejunum and ileum, as in our case, are more likely to be metastatic at the time of diagnosis because they are often asymptomatic and difficult to detect during their early, local-regional stage [14]. Prognosis of SBNETs correlates with the staging of the tumor. For well-differentiated SBNETs with local-regional spread like our patient’s case, the median survival is 111 months and decreases to 56 months in metastatic disease [16].

According to the 2024 European Neuroendocrine Tumor Society (ENETS) guidelines, all localized, resectable SBNET should undergo curative resection using an open approach with bimanual palpation of the entire small bowel. This is combined with a vessel-sparing lymphadenectomy (at least >8 lymph nodes) limiting the extent of small bowel resection. Due to our patient’s advanced age and local-regional disease, a minimally invasive, laparoscopic approach was used in our case. This approach is still controversial, but is increasingly accepted at high-volume centers, as it has shown similar results in terms of the number of resected lymph nodes and long-term outcomes. Current evidence does not support the use of adjuvant therapy after curative resection, only routine follow-up as in our case [4].

Somatostatin analogs (SSAs) are the treatment of choice in carcinoid syndrome. SSAs are also the first-line therapy in grade 1 or 2 advanced, unresectable SBNETs. SSAs have been shown to delay disease progression and improve progression free survival compared to placebo in early grade, midgut NETs expressing somatostatin receptors (SST) with a Ki-67 proliferation index less than 10% [4]. In cases where there is evidence of rapid disease progression (Ki-67 index >10%) or high tumor burden, more intense treatments with everolimus, peptide receptor radionuclide therapy (PRRT), or systemic chemotherapy are often considered. However, data beyond first-line SSA is still lacking, and individualized care with multidisciplinary approach is critical to management [4].

## Conclusions

In conclusion, SBNETs are an uncommon cause of obscure or small bowel bleeding that have been increasing in incidence and prevalence over the last few decades. Early-stage SBNETs are difficult to detect due to their indolent nature. As such, clinicians should consider SBNETs in the differential when patients present with nonspecific abdominal symptoms including obscure GI bleeding. When small bowel GI bleeding is suspected, screening VCE should be performed early to help facilitate subsequent DBE. Combining VCE and DBE is not only safer and more tolerable for patients, but is critical for the timely diagnosis and treatment of SBNETs.
